# Impact of an Interprofessional Collaboration Between Physicians and Pharmacists on Fall-Risk-Increasing Drugs in Older Patients with Trauma in the Emergency Department

**DOI:** 10.3390/geriatrics10020046

**Published:** 2025-03-17

**Authors:** Benjamin J. Hellinger, André Gries, Thilo Bertsche, Yvonne Remane

**Affiliations:** 1 Emergency Department, Observation Unit, Leipzig University Medical Center, 04103 Leipzig, Germany; 2 Pharmacy, Leipzig University Medical Center and Medical Faculty, 04103 Leipzig, Germany; 3 Drug Safety Center, Leipzig University and Leipzig University Medical Center, 04103 Leipzig, Germany; 4 Clinical Pharmacy, Institute of Pharmacy, Medical Faculty, Leipzig University, 04103 Leipzig, Germany

**Keywords:** adverse drug reaction, clinical pharmacist, clinical pharmacy services, deprescribing, emergency department, falls, fall-risk-increasing drugs, polypharmacy

## Abstract

**Background/Objectives**: In older patients, falls constitute a significant public health concern and a major cause of hospital admission. Fall-risk-increasing drugs (FRIDs) represent a key risk factor for falls. Therefore, modifying these drugs represents an important strategy for preventing recurrent falls and further patient harm. The objective of this study was to evaluate a structured interprofessional collaboration between physicians and pharmacists on managing FRIDs in older patients who present to the emergency department (ED) after a fall. **Methods**: This study was performed in the ED of a tertiary care hospital. Patients who were >65 years old and presented to the ED after a fall were included. A routine care group was included between 1 March 2020 and 31 May 2020. A pharmaceutical care group was included between 1 September 2023 and 30 November 2023. In the pharmaceutical care group, a clinical pharmacist supported the physicians in identifying and managing FRIDs. Possible solutions for improving FRID prescription were discussed interprofessionally. The number of FRIDs at ED admission and discharge, as well as the number of FRID modifications, were evaluated. **Results**: A total of 107 patients were enrolled in each group. There were 85 patients in the routine care group and 89 patients in the pharmaceutical care group, with at least 1 FRID prescribed at ED admission (*p* = 0.483). At ED discharge, there were 85 patients in the routine care group and 68 patients in the pharmaceutical care group, with at least 1 FRID prescribed at (*p* = 0.010). There were seven FRID modifications in the routine care group compared to 125 FRID modifications in the pharmaceutical care group. **Conclusions**: In this study, the interprofessional collaboration between physicians and pharmacists led to a reduced number of FRIDs being prescribed and more FRID modifications in older patients at ED discharge. Further research is required to ascertain the feasibility of integrating this single intervention into a multifactorial fall prevention program.

## 1. Introduction

Falls in older patients pose a major challenge to society and are becoming more prominent due to demographic changes [1]. Approximately 35% of persons aged 65 or older fall at least once a year; for nursing home residents, the incidence is considered to be 50% [2]. Data from the American Centers for Disease Control estimate that, after a fall, around 3 million older adults present to the emergency department (ED) in the USA per year, imposing a major burden for the healthcare system [3]. Around a fourth of falls in older patients require hospital treatment [4]. Serious injuries such as intracranial bleeding or hip fractures can lead to long-term health impairments. In addition to the health damage caused by falls for older patients, psychological and social consequences can also arise. A fear of falling again reduces their own range of movement and can lead to social isolation [5,6].

Therefore, preventing falls constitutes one of the main goals of relieving the burden for patients and healthcare systems. Many different risk factors for falling exist, and these can and should be addressed via interventions. These risk factors are often categorized into extrinsic risk factors (e.g., uneven floors or bad lighting) and intrinsic risk factors (e.g., orthostatic hypotension or impaired mobility). A multifactorial risk factor assessment and corresponding individualized interventions on a patient-by-patient basis are recognized as pivotal elements for the prevention of recurrent falls and patient harm [7,8,9]. Such interventions may include physiotherapy or occupational therapy. One aspect that should be addressed in this multifactorial risk assessment is the patient’s medication. When prescribing and administering drugs that increase the risk of falling, especially in the context of increasing polypharmacy, so-called fall-risk-increasing drugs (FRIDs) constitute an intrinsic risk factor [10,11]. Recent studies have reported that over 50% of older patients are prescribed FRIDs [12,13]. As falls are a multifactorial phenomenon and often not measured as an adverse drug reaction (ADR) in pharmacovigilance studies, it remains challenging to definitively identify a drug-related causal relationship. Comprehensive data on falls caused by drugs are increasingly being explored but have not yet been conclusively analyzed [5].

The indications for drugs that are considered FRIDs are very heterogeneous, but the mechanisms that lead to an increased risk of falls are often similar [5]. On the one hand, some FRIDs can impair patients’ cognitive abilities by depressing the central nervous system (CNS) (e.g., benzodiazepines and antidepressants) [14]. The depression of the CNS can limit individuals’ perception of the environment: for example, individuals’ awareness of uneven surfaces decreases and undesirable side effects such as dizziness impair coordination [15]. On the other hand, some FRIDs, intentionally or unintentionally, affect the cardiovascular system and can lead to orthostatic dysregulation or chronotropic insufficiency. These ADRs increase the risk of (pre-)syncopal events with subsequent falls [16]. Geriatric patients are more susceptible to ADRs due to limited drug elimination, an altered distribution volume, and a sometimes slower metabolism. Given the heightened risk of ADRs in this patient population, it is imperative to closely monitor the incidence and severity of those ADRs [17].

The ED is often the initial point of contact for older patients who have fallen. It is important to recognize that ED physicians frequently have limited time to comprehensively evaluate and adjust medications that may contribute to the risk of falls [18]. Their primary responsibility is to address the immediate trauma resulting from the fall, which can limit opportunities for in-depth medication management. But the ED is also an opportunity to address FRIDs at an early stage in the patient’s rehabilitation after a fall. Despite an increasing awareness of this problem, it is often difficult to reduce FRIDs and, thus, the problem is not routinely addressed [12]. With increasing polypharmacy and more complex medication regimens, physicians face growing challenges in avoiding medication errors, underscoring the need for specialized oversight. This domain could become an area of responsibility for clinical pharmacists, who are already managing medication successfully in a variety of clinical settings [19,20,21,22]. In the ED, addressing the identification of FRIDs in older ED patients and establishing strategies for reducing or improving FRID use in interdisciplinary teams might be one way for clinical pharmacists to contribute to improved medication safety. Once identified, FRIDs could be deprescribed or replaced by safer alternatives if feasible, in collaboration with the treating physicians. For example, alpha-blockers for hypertension can cause significant orthostatic dysregulation, leading to falls, and might be replaced with drugs more suitable for older patients, such as ACE inhibitors. If no alternatives are available, adjusting the dose or using other drug formulations might offer the opportunity to reduce the occurrence of ADR and subsequent falls.

The objective of this study was therefore to ascertain the impact of an interprofessional collaboration between physicians and pharmacists on FRIDs in older patients with trauma in the ED. To this end, we integrated a clinical pharmacist into the interdisciplinary ED team, with a particular focus on FRID identification and FRID management.

## 2. Materials and Methods

### 2.1. Study Setting

This study was performed in the ED of a university hospital offering tertiary care. The hospital has a capacity of around 1450 beds and the ED treats approximately 2500 patients per month. The ED treats adults ≥18 years and is staffed with a core team of long-term ED physicians and additional physicians from the departments of surgery, internal medicine, and neurology, as well as nurses specialized in emergency medicine.

### 2.2. Patients

Consecutive patients presenting to the ED between 8:30 a.m. and 1:30 p.m. on weekdays were enrolled. This study included patients 65 years of age or older who presented to the ED for a fall within the previous 24 h. Patients who came to the ED by themselves and those who arrived by ambulance were included. Patients were screened for chief complaints according to the Manchester Triage System potentially associated with a fall: pain/trauma limb, pain/trauma torso, fall, wound, head injury, and major trauma. Falls resulting from external factors (e.g., traffic accident) were excluded. Patients that returned to the ED for secondary problems due to a previous fall (e.g., persistent pain) were excluded. Patients transferred from another hospital or directly transferred to the intensive care unit (ICU) were also excluded.

### 2.3. Study Design

This study was a consecutive, controlled intervention study that investigated FRID prescription in older patients presenting to the ED after a fall and how the ED team manages those FRIDs. The study protocol was approved by the responsible Ethical Committee and the scientific evaluation was conducted anonymously. The routine care group and pharmaceutical care group were assessed consecutively in order to avoid learning effects of the pharmaceutical intervention in the routine patients. The implementation of a clinical pharmacist within the ED was initially promoted by the leading physician of the ED. After implementation, the clinical pharmacist was identified as a key factor in the optimization of care for geriatric patients. The design and execution of the project was developed between the ED, pharmacy and Department of Clinical Pharmacy.

#### 2.3.1. Routine Care Group

The routine care group was included between 1 March 2020 and 31 May 2020. Patients were treated for trauma and injuries by physicians from the surgical department in the ED. The patients’ medical history, drug history, and laboratory results were obtained by the physicians. If the treating physician had modified or recommended modifying the patients’ drug therapy regarding FRIDs, the information was documented in the discharge letter, for inpatients and outpatients, respectively. During this period, no clinical pharmacy services were established directly in the ED. A drug information unit run by the pharmacy was available to all physicians at the hospital via telephone.

#### 2.3.2. Pharmaceutical Care Group

The pharmaceutical care group was included between 1 September 2023 and 30 November 2023. The initiation of the pharmaceutical care group was delayed due to the COVID-19 pandemic, which resulted in contact restrictions and the redeployment of study personnel to other critical tasks. A clinical pharmacist was integrated into the ED team and conducted a targeted medication review for patients after a fall. The medication review was performed with a particular focus on identifying FRIDs. For FRID identification, a list was drawn up from the Beers Criteria and PRISCUS list; this included all drugs associated with either a fall or orthostatic dysregulation [23,24]. An extended medication history was obtained by the clinical pharmacist, if necessary (e.g., through relatives or general practitioner).

After identifying the FRIDs, the clinical pharmacist worked out possible solutions for drug management and discussed them with the treating physician. In this structured interprofessional discussion, the potential FRIDs were analyzed and appropriate alternatives or adjustments were considered. The jointly agreed-upon decision regarding FRID management was documented in the discharge notes, for inpatients and outpatients, respectively. This was communicated to the ward providing further treatment or given to the patient to discuss with their general practitioner. If no modification was made because the benefit of the drug’s effect outweighed the risk of a fall, in the interprofessional discussion, this was documented as “no modification performed”. This procedure was applied directly in the ED as part of the routine treatment process in order to avoid longer delays in patient care.

### 2.4. Data Collection

The following data were taken from the patient’s electronic medical record at the hospital: demographic parameters (age, sex), the patient’s medication (including drug, dose and administration time, number of drugs taken) and the reason for falling. The reason for falling was classified as stumbling fall, (pre-)syncopal fall, and fall of unknown reason. The number of FRIDs taken was assessed at ED admission and at ED discharge. Any modifications to FRID therapy that the physician initiated in the routine care group or performed as a result of the interprofessional discussion in the pharmaceutical care group were derived from the medical record. Multiple drug therapy modifications were possible in one patient. The modifications were classified into the following groups (based on PCNE classification of drug-related problems): drug stopped, drug paused, dose changed, administration time changed, alternative drug prescribed, further clarification with general practitioner recommended, and no modification made [25]. All collected data were anonymized and electronically documented in a database in Microsoft Excel.

### 2.5. Data Analysis

The number of patients with at least 1 FRID prescribed at ED admission and ED discharge were compared between the routine care group and pharmaceutical care group. The median number of FRIDs per patient at ED admission and at ED discharge between the routine care and pharmaceutical care group were evaluated. The number of drug therapy modifications on FRIDs performed in the ED were compared. Furthermore, age, sex, and reason for falling were compared. For the following scientific evaluation, FRIDs were classified into groups according to the STOPPFall criteria for the routine care and pharmaceutical care group [26]. For metric and ordinal variables, the median and IQR were calculated. The Mann–Whitney U test and Chi-squared test were performed using IBM SPSS Statistics 27.0. The results are given as two-sided *p*-values. *p* ≤ 0.05 was considered statistically significant.

## 3. Results

**Figure 1:**
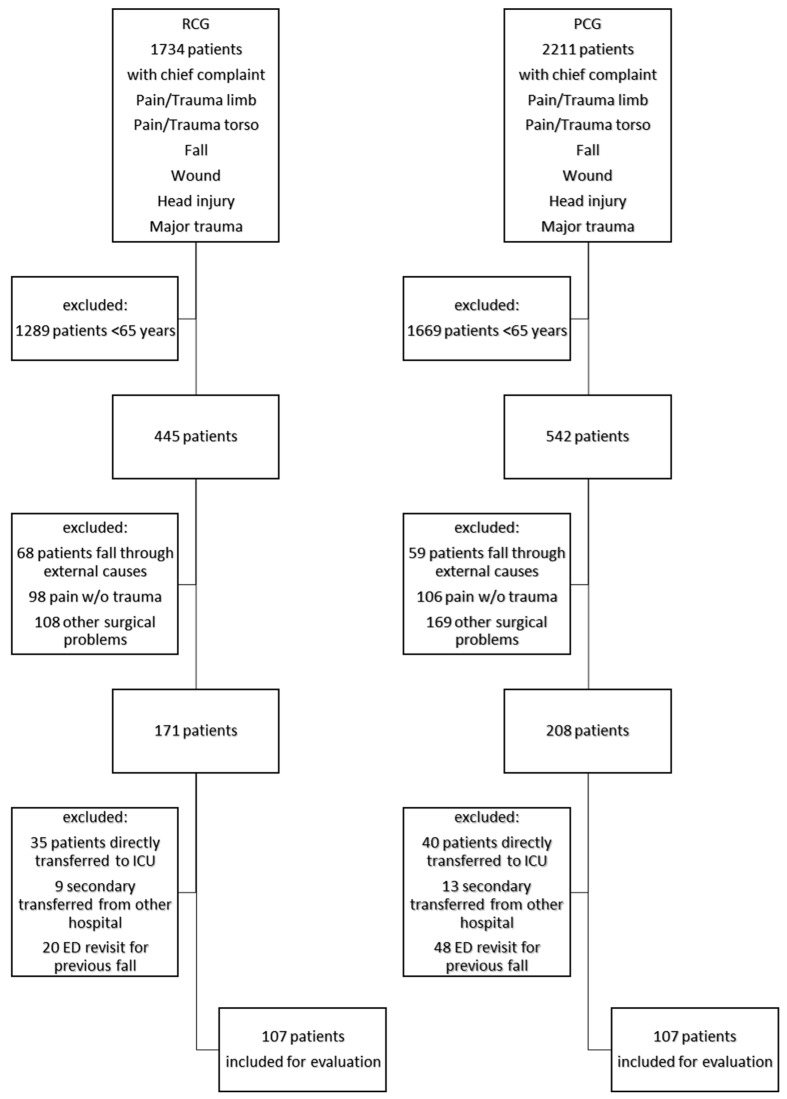
Patient inclusion process in routine care group (RCG) and pharmaceutical care group (PCG); fall through external cause, e.g., traffic accident.

In total, 107 patients were included in the routine care group. In the pharmaceutical care group, 107 patients were also included (Figure 1).

### 3.1. General Patient Characteristics

There were no major differences in baseline characteristics such as the patient’s sex, the number of drugs taken, and the intake of FRIDs between the routine care group and the pharmaceutical care group. Patients in the pharmaceutical care group tended to be older. The results are displayed in Table 1.

In the routine care group, a total of 218 FRIDs were documented at ED admission. In the pharmaceutical care group, a total of 220 FRIDs were documented. The most common classes of drugs were diuretics in both groups, accounting for approximately a fourth of FRIDs. The FRIDs prescribed to patients who presented with a fall in the previous 24 h are displayed in Table 2.

### 3.2. Patients with FRID Prescription at ED Discharge

There were 85 patients in the routine care group and 89 patients in the pharmaceutical care group who had at least one FRID prescribed at ED admission. There was no statistically significant difference between the groups according to the chi-squared test (*p* = 0.483). At ED discharge, there were still 85 patients with at least one FRID prescribed in the routine care group, compared to only 68 patients in the pharmaceutical care group. This difference was statistically significant according to the chi-squared test (*p* = 0.010).

### 3.3. Number of FRIDs at ED Discharge

In the routine care group, the median number of FRIDs per patient at ED admission was two (1–3) (median IQ25, IQ75) in the pharmaceutical care group. There was no statistically significant difference between the groups according to the Mann–Whitney U test (*p* = 0.892). At ED discharge, the median number of FRIDs per patient was 2 (1–3) in the routine care group, compared to and 1 (0–2) in the pharmaceutical care group. This difference was statistically significant according to the Mann–Whitney U test (*p* = 0.003).

### 3.4. Drug Therapy Modifications

The physicians made a total of seven drug therapy modifications in seven patients in the routine care group. In total, 78 of the 85 patients taking at least one FRID received no drug therapy modification in the routine care group. Compared to that, in the pharmaceutical care group, drug therapy modifications were made for 125 of the 220 identified FRIDs for 83 patients. The number of modifications ranged between one and three per patient. In total, 6 of the 89 patients taking at least one FRID received no drug therapy modification in the pharmaceutical care group. After the interprofessional discussion of the risk–benefits of FRID use, no modifications were made for 95 of the 220 identified FRIDs in the pharmaceutical care group. The results are displayed in Table 3.

## 4. Discussion

The findings of this study demonstrate that the interprofessional collaboration of physicians and clinical pharmacists in the ED can improve the effective identification and reduction in the prescription of FRIDs in older patients who have experienced a fall. As a result of this collaboration, the number of patients with a FRID prescription decreased significantly at ED discharge with pharmaceutical care. Furthermore, the number of FRIDs per patient was reduced by one FRID at the time of ED discharge in the pharmaceutical care group in comparison to no reduction in the routine care group. In addition to the reduction in the prescription of FRIDs by stopping or pausing drugs, which is not always a viable option, other drug therapy modifications were employed to improve FRID prescription. These changes included adjustments to dosage or the timing of drug administration. The number of FRID therapy modifications increased around 10-fold in the pharmaceutical care group (7 vs. 125). Based on the risk–benefit assessment, however, a considerable number of FRIDs were not modified after the interprofessional discussion, despite an identification (95 out of 220 FRIDs).

### 4.1. FRID Classes

The type of FRIDs prescribed was found to be similar between the routine care and pharmaceutical care groups, with the majority of prescribed FRIDs being diuretics in both groups. Patients with those FRIDs often presented with hypotension or hypovolemia, which can predispose them to syncopal events and subsequent falls. Additionally, sn electrolyte imbalance caused by diuretics can also contribute to an increased risk of falls. Furthermore, antihypertensive or urological agents (alpha-receptor blockers) can cause orthostatic dysregulation, for example, and these were frequently identified as FRIDs.

A significant number of centrally acting drugs, which are known to cause CNS depression, were prescribed and identified as FRIDs in both groups. Patients indicated that dizziness or an unsteady gait was the underlying cause of their falls. These symptoms may be attributable to CNS-depressing drugs, but they may also be indicative of pre-existing illnesses. It is well documented that these medications can impair cognitive abilities in older patients; however, they are still widely prescribed to address the symptoms of aggression associated with dementia [27].

### 4.2. FRID Management

Over three-fourths of the individuals who presented to the ED after a fall were taking at least one FRID. The identification of these FRIDs could reduce the risk of falls by implementing appropriate medication management strategies.

The number of drug modifications to FRID therapy increased around tenfold in the pharmaceutical care group through interprofessional collaboration compared to the routine care group. In particular, stopping drugs that were no longer indicated and adjusting doses were much more frequent in the pharmaceutical care group.

Diuretics were frequently discontinued. This is because the patients often had a hypovolemic volume status, which can be detected in the ED with little effort. Discontinuation was particularly common if they were prescribed diuretics for a high blood pressure. If necessary, alternatives that are safer for geriatric patients such as ACE Inhibitors or calcium channel blockers were prescribed. In patients treated with loop diuretics for the treatment of edema in heart failure, the discontinuation of these FRIDs can be more challenging as it increases the risk of exacerbating the underlying disease. Additionally, electrolyte imbalances, particularly hyponatremia, were observed in some patients as a side effect of diuretic therapy. However, the susceptibility of geriatric patients to diuretic side effects is often overlooked when prescribing them [28,29]. Therefore, diuretics should only be used with caution in older patients due to their potential ADR, especially for antihypertensive indications, and replaced with more suitable alternatives.

In patients for whom drugs such as tamsulosin, which can cause orthostatic dysregulation when taken in the morning, or mirtazapine, which can cause daytime sleepiness, were prescribed, the administration times were adjusted in the pharmaceutical care group. Similarly, the dosage of opioids was frequently reduced if the patients reported prolonged pain freedom and agreed to a reduction. In addition, a subset of patients received high doses of opioids without concurrent non-opioid therapy, despite this being recommended by the WHO analgesic ladder [30]. For those patients, the introduction of non-opioid therapies could help to reduce opioid prescriptions while keeping patients free of pain. However, the data on reducing the risk of falls by adjusting dosages are conflicting [31].

In conclusion, most FRID modifications involved the following drug classes: diuretics, opioids, urological agents, and antihypertensives. In the ED, the conditions these medications treat can be assessed by the staff with little effort (e.g., electrolytes, pain, blood pressure or volume status), enabling timely adjustments to drug therapy.

In contrast, the drugs prescribed for neurologic or psychiatric diseases seem to be harder to address, since the interdisciplinary discussion in the pharmaceutical care group often led to no FRID modification (95 out of 220 FRIDs). The risk–reward evaluation of those FRIDs often resulted in the acceptance of a potentially increased fall risk in favor of a well-treated more complex disease (e.g., Parkinson’s disease, depression or epilepsy). Nevertheless, identifying and developing awareness of a FRID alone should facilitate the reevaluation of its prescription if falls recur.

### 4.3. Summary

In conclusion, the present study offers promising evidence that reducing the intake of FRIDs through interprofessional collaboration is feasible in the setting of the ED and may present one way to improve patient safety. The clinical pharmacist enhanced interprofessional collaboration by collecting key data on FRID use and potential ADRs through direct patient interviews and by closely collaborating with physicians on FRID management, leading to improved medication safety. However, it is important to recognize that this intervention represents only one component of a broader, multifactorial and multiprofessional strategy to mitigate the risk of falls. Future research should focus on determining how this single measure can be effectively integrated into comprehensive risk assessment frameworks, ensuring that it complements other preventive efforts to achieve a more robust reduction in the incidence of falls. Achieving this objective in an effective manner would enhance the quality of life for older patients, e.g., promoting enhanced mobility, reducing hospitalizations, and improving independence.

### 4.4. Limitations

Although FRID prescription was significantly reduced in this study, we cannot conclude that there were fewer subsequent falls in the pharmaceutical care group due to the lack of a follow-up program. Nor could any potentially negative impacts of discontinuing FRIDs be assessed. Furthermore, the decisions concerning drug therapy changes reached between the physician and pharmacist in the ED might not be implemented by the general practitioner in the outpatient setting. Therefore, this study does not allow for any conclusions regarding the integration of the intervention into the comprehensive process of fall reduction for patients. As patients were only included at a defined daytime, falls that happened during the night may have been underreported or represent a selection bias in the routine care and pharmaceutical care groups. It should also be noted that the results of this study might not be generalizable to all older patients, as a significant proportion of this demographic does not visit an ED after a fall and their FRID prescription might differ. Patients in the pharmaceutical care group were significantly older by a median of 3 years; therefore, they might be more prone to falls in the first place. However, this difference may not be clinically relevant for this study as the FRID distribution, FRIDs prescribed, and number of drugs taken did not differ between the groups. Furthermore, the three-year interval between the routine care and intervention groups could have created bias. However, no specific workflows or staff training for FRID management had been established in the ED prior to the work of the clinical pharmacist in this study, therefore minimizing the risk of bias.

## 5. Conclusions

Involving a clinical pharmacist in the interprofessional treatment team of the ED contributes to identifying FRIDs and developing feasible management strategies. Through this interprofessional collaboration, the prescription of FRIDs significantly decreased. An appropriate clinical management strategy involving the discontinuation, pausing, or dose adjustment of any concerning medication could be developed. In the future, studies that promote the long-term implementation of these suggestions in routine care and lead to a decrease in falls at the patient level should be performed.

## Figures and Tables

**Table 1:** Baseline characteristics of the routine care group and pharmaceutical care group.

	Routine Care Group	Pharmaceutical Care Group	*p*-Value
No. of patients included	107	107	
Sex % (n)			0.492
Female	58 (62)	62 (66)	
Male	42 (45)	38 (41)	
Age median (Q25–Q75)	82 (76.5–87)	85 (80–90)	0.006
No. of drugs taken, median (Q25–Q75)	7 (4–9)	7 (5–10)	0.608
No. of Patients with at least 1 FRID at ED admission (%)	85 (79.4)	89 (83.2)	0.483
No. of FRIDs/patient median (Q25–Q75) At ED admission	2 (1–3)	2 (1–3)	0.892

FRIDs, fall-risk-increasing drugs; *p*-values calculated with Mann–Whitney U test/Chi-squared test.

**Table 2:** Fall-risk-increasing drugs at ED admission (based on STOPPFall classification [26]).

Drug Class	Routine Care Group *n* = 218 (%)	Pharmaceutical Care Group *n* = 220 (%)
Diuretics	55 (25.2)	61 (27.7)
Neuroleptics	31 (14.2)	33 (15.0)
Antidepressants	24 (11.0)	25 (11.4)
Antiepileptics	21 (9.6)	23 (10.5)
Opioids	32 (14.7)	22 (10.0)
Urologics/Anticholinergics	15 (6.9)	19 (8.6)
Parkinson disease drugs	5 (2.3)	11 (5.0)
Antihypertensives	15 (6.9)	7 (3.2)
Sedatives/Benzodiazepines	6 (2.8)	6 (2.7)
Antihistamines	0 (0.0)	4 (1.8)
Others	14 (6.4)	9 (4.1)

**Table 3:** Fall-risk-increasing drugs (FRIDs) and their modification at ED discharge.

	Dose Adjusted	Drug Stopped	Drug Paused	Alternative Drug Prescribed	Administration Time Changed	Further Clarification with GP Recommended	No Modification
Diuretics							
PCG	9	23	1	6	0	1	21
RCG	0	1	2	0	0	0	52
Neuroleptics							
PCG	1	1	0	0	2	0	29
RCG	0	1	0	0	0	0	31
Antidepressants							
PCG	2	3	0	0	1	3	16
RCG	0	1	0	0	0	0	24
Antiepileptics							
PCG	3	3	0	0	0	5	12
RCG	0	0	0	0	0	0	21
Opioids							
PCG	9	2	0	8	0	0	3
RCG	0	1	0	0	0	0	32
Urologic agents							
PCG	0	0	0	4	9	0	6
RCG	0	0	0	0	0	0	15
Antihypertensives							
PCG	0	3	2	1	0	1	0
RCG	0	0	0	0	0	0	15
Parkinson drugs							
PCG	0	0	0	0	0	0	11
RCG	0	0	0	0	0	0	5
Sedatives							
PCG	2	2	0	1	0	0	1
RCG	0	0	0	0	0	0	6
Antihistamines							
PCG	0	3	0	0	0	0	1
RCG	0	0	0	0	0	0	0
Others							
PCG	0	8	0	0	0	1	0
RCG	0	1	0	0	0	0	14

PCG—pharmaceutical care group; RCG—routine care group; GP—general practitioner.

## Data Availability

Data are available from the authors upon reasonable request.

## Abbreviations

The following abbreviations are used in this manuscript:

ADR	Adverse drug reaction
CNS	Central nervous system
ED	Emergency department
FRID	Fall-risk-increasing drug
ICU	Intensive care unit
PCG	Pharmaceutical care group
PCNE	Pharmaceutical Care Network Europe
RCG	Routine care group
